# SAFE AND SENSATIONAL: ASSESSING THE SECURE AND PRIVATE ONLINE DATING WORLD FOR OLDER ADULTS

**DOI:** 10.1093/geroni/igad104.0869

**Published:** 2023-12-21

**Authors:** Naheem Noah, Sanchari Das

**Affiliations:** University of Denver, Denver, Colorado, United States; University of Denver, Denver, Colorado, United States

## Abstract

Online dating applications have become a popular way for people worldwide to meet potential partners, including older adults aged 65 and above. However, using these applications involves revealing personally identifiable information (PII), which raises privacy and security concerns, particularly for older adults who are often the targets of cyberattacks. To address this issue, this paper conducted a literature review of 38 peer-reviewed publications on online dating security and privacy, with a focus on older adults. The review found that while 10 papers evaluated older adults’ cybersecurity issues, and two papers explored mobile security management with adults, no papers explicitly focused on older adults and dating applications. To fill this research gap, this paper conducted an application-level security and privacy analysis of ten popular online dating applications, including Tinder, Grindr, Match, Bumble, OkCupid, SilverSingles, Harmony, EliteSingles, Christian Mingles, and LDS Singles. The study revealed that these applications have concerning data security practices that may affect the privacy and security of users, especially older adults. The paper concludes by providing actionable recommendations for secure and privacy-preserving user experience. For instance, online dating applications should implement secure authentication mechanisms, such as two-factor authentication, and use end-to-end encryption to protect users’ data. Additionally, the applications should enable users to control their data and allow them to choose the information they want to share with others. Moreover, the dating apps should provide clear and concise privacy policies that are easy to understand and readily available to users.

